# Pancreatoduodenectomy versus total pancreatectomy and simultaneous intraportal islet autotransplantation for periampullary cancer at high-risk of postoperative pancreatic fistula (XANDTX-trial): Protocol of a randomized controlled pilot trial

**DOI:** 10.1371/journal.pone.0327949

**Published:** 2025-07-28

**Authors:** Sebastian Hempel, Fiona R. Kolbinger, Florian Oehme, Olga Radulova-Mauersberger, Janine Schmid, Undine Schubert, Florian Schepp, Stefan Bornstein, Sandra Korn, Evelyn Trips, Jürgen Weitz, Marius Distler, Barbara Ludwig

**Affiliations:** 1 Department of Visceral, Thoracic and Vascular Surgery, University Hospital and Faculty of Medicine Carl Gustav Carus, Technische Universität Dresden, Dresden, Germany; 2 National Center for Tumor Diseases (NCT/UCC), Dresden, Germany; 3 German Cancer Research Center (DKFZ), Heidelberg, Germany; 4 Faculty of Medicine and University Hospital Carl Gustav Carus, Technische Universität Dresden, Dresden, Germany; 5 Helmholtz-Zentrum Dresden - Rossendorf (HZDR), Dresden, Germany; 6 Department of Medicine III, University Hospital and Faculty of Medicine Carl Gustav Carus, Technische Universität Dresden, Dresden, Germany; 7 Paul Langerhans Institute Dresden of the Helmholtz Center Munich, University Hospital and Faculty of Medicine Carl Gustav Carus, Technische Universität Dresden, Dresden, Germany; 8 Center for Diabetes Research (DZD e.V.), Neuherberg, Germany; 9 Division of Diabetes & Nutritional Sciences, School of Cardiovascular and Metabolic Medicine & Sciences, King’s College London, London, United Kingdom; 10 Coordination Centre for Clinical Trials, Faculty of Medicine Carl Gustav Carus, TUD Dresden University of Technology, Dresden, Germany; PLOS: Public Library of Science, UNITED KINGDOM OF GREAT BRITAIN AND NORTHERN IRELAND

## Abstract

**Introduction:**

Pancreatic surgery remains associated with significant morbidity. Pancreatoduodenectomy (PD) with high-risk stigmata for postoperative pancreatic fistula (POPF) may delay or hinder administration of adjuvant therapy. Total pancreatectomy (TP) prevents POPF-associated complications but implies permanent exocrine and endocrine insufficiency. Islet autotransplantation (IAT) has the potential to compensate endocrine function.

**Methods and analysis:**

XANDTX is a single-centre randomized controlled pilot trial comparing high-risk PD with TP and simultaneous IAT in patients with periampullary cancer. After screening for eligibility and obtaining informed consent, a total of 32 adult patients will be intraoperatively randomized in a 1:1 ratio. The primary hypothesis is that TP with IAT prevents POPF-associated complications and leads to a shorter period to initiation of adjuvant therapy and a higher overall rate of adjuvant therapy administration.

Secondary endpoints include perioperative morbidity and mortality, metabolic outcome, quality of life (QoL) and oncological long-term outcome. Each patient will be followed up for 5 years.

**Discussion:**

The XANDTX pilot trial will aim to provide surgical and oncological feasibility and safety data of total pancreatectomy with simultaneous islet autotransplantation in management of resectable periampullary cancer. The results will form the basis for a further confirmatory controlled study.

**Trial registration:**

This study was registered on ClinicalTrials.gov (NCT05843877) on February 27, 2023 and EudraCT (2023-507773-17-00) on April 18, 2024.

## Introduction

Despite steady improvement in surgical technique, optimization of perioperative management and increasing centralization, pancreatic surgery remains associated with significant morbidity. Although mortality could be significantly reduced, morbidity is still reported at a rate of 40–60%, even in high-volume centers [1]. Especially for pancreatic malignancies, tumor resection represents the only potentially curative therapeutic procedure. One of the most common and potentially life-threatening complications in pancreatic surgery is postoperative pancreatic fistula (POPF), occurring at varying frequency depending on the type and extent of pancreatic resection or intervention. Pancreatic anastomosis in pancreaticoduodenectomy (PD) represents an “Achilles heel” in pancreatic surgery as leakage or insufficiency of the pancreaticoenteric anastomosis can result in POPF and POPF-associated complications. Over the last decades, many studies have analyzed POPF risk factors, with the strongest evidence for a considerably increased prevalence of POPF in patients with soft pancreatic parenchyma and a small pancreatic duct diameter [2–4].

Since total pancreatectomy (TP) avoids a pancreaticoenteric anastomosis and therefore circumvents POPF-associated complications, TP has recently become a major topic of scientific discussion in the surgical community as a potential alternative to partial pancreatic resection in patients with a high-risk constellation. In the treatment of patients with periampullary cancers, primary TP provides surgical and oncological outcomes similar to standard pancreatic head resection [5]. Recently published studies compared the surgical outcomes of high-risk PD and TP, often favouring TP [6–8]. A retrospective analysis also reported on generally comparable nonspecific, cancer-specific, and pancreas-specific quality of life (QoL) between TP and high-risk PD [9]. However, the psychosocial impact of complete insulin-deficient and often brittle diabetes in combination with severe exocrine insufficiency were significantly pronounced after TP, emphasising the life-long burden related to a complete removal of pancreatic parenchyma. Intraportal islet autotransplantation (IAT) simultaneous to TP is a potential option to prevent or alleviate pancreoprivic diabetes through partial preservation of endogenous insulin secretion [10–12]. Specifically, pancreatic islets are enzymatically isolated from the non-tumor-bearing part of the pancreas after TP and are re-infused via the portal vein, resulting in intrahepatic engraftment [13,14]. IAT thereby has a significant positive effect on metabolic control and QoL [15–18].

The objective of the XANDTX trial is to obtain data on the treatment effect and safety of combined TP and simultaneous IAT in patients with periampullary carcinoma at high-risk for POPF. The feasibility and safety data regard both surgical and oncological outcome comparing to the current surgical standard procedure.

## Methods and analysis

### Trial design and timeline

**Fig 1:**
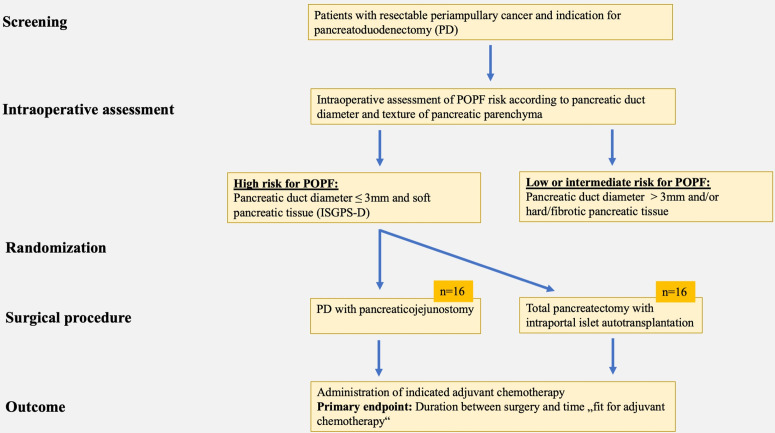
Study flowchart.

XANDTX is conceptualized as a single-centre open-label, randomized, controlled therapeutic exploratory feasibility study. Patients planned for elective periampullary tumor resection will be assessed for eligibility and eligible patients will be informed about the trial in detail. After written informed consent and in the case of intraoperative presence of a POPF high-risk constellation (soft pancreatic texture and pancreatic duct diameter ≤ 3 mm), 32 patients will be intraoperatively allocated in a 1:1 ratio to undergo either PD or TP with simultaneous IAT (Fig 1). Randomization will be performed via sealed envelopes (block randomization).

The recruitment start for this study was in January 2025. The planned recruitment period is 24 months. The individual follow-up time after surgery is 60 months, resulting in a study duration of 84 months, after which results are expected. The trial will be reported according to the Consolidated Standards of Reporting Trials (CONSORT) Extension for Randomized Pilot and Feasibility Trials [19].

### Population and eligibility criteria

In this study, 32 patients with periampullary carcinoma and high-risk profile for the occurrence of POPF will be treated. Inclusion of minors and non-consenting subjects is not intended. All in- and exclusion criteria are listed in Table 1. A balanced gender distribution in the study population is aspired. However, the determination of possible sex-specific differences in efficacy or safety of the treatment arms will likely be impossible due to the small case number and must be investigated in larger follow-up studies. The Department of Visceral, Thoracic and Vascular Surgery at the University Hospital Carl Gustav Carus Dresden has a focus on oncological pancreato-biliary surgery, performing about 150 pancreatic resections (including at least 70 PDs) annually. Approximately 20 of 70 cases of pancreatic head resections meet the criteria of a high-risk profile for POPF. Therefore, recruitment of 16 patients/year appears feasible, resulting in a planned recruitment duration of 24 months.

**Table 1:** Inclusion and exclusion criteria.

Inclusion criteria	Exclusion criteria
• suspected or histologically confirmed periampullary carcinoma/tumor and indication for PPPD or Whipple surgery • high-risk constellation for the development of POPF: soft pancreas and pancreatic duct diameter ≤ 3 mm • male and female patients aged 18 years and older • written informed consent	• patients who are undergoing simultaneous surgery in addition to PPPD or Whipple surgery • expected lack of adherence • histologically confirmed other primary tumor • previous transplantation of an organ or tissue • known infection with HIV (HIV antibodies) • positive hepatitis C antibodies, positive hepatitis B surface antigens and hepatitis Bc antibodies • insulin-deficient diabetes mellitus • pregnant or breastfeeding women • participation of the patient in another clinical trial according to AMG within the last 4 weeks before inclusion

Abbrevations: AMG, Arzneimittelgesetz (German Medicinal Products Act); HIV, human immunodeficiency virus; PD, pancreaticoduodenectomy; POPF, postoperative pancreatic fistula.

### Ethics dissemination

The present trial will be conducted in accordance with the Declaration of Helsinki, the international principles of GCP (ICH-GCP E6 guidelines), the provisions of the AMG and the GCP Regulation (2004) and all other applicable laws including data protection laws and the European General Data Protection Regulation. The study protocol (version 6.0F, March 27, 2024) was reviewed and approved by the responsible federal authority (Paul-Ehrlich-Institut (Agency of the German Federal Ministry of Health)) and the responsible ethics committee through the EU portal in accordance with the Regulation (EU) 536/2014 (EUCT-number: 2023-507773-17-00).

Each included patient will be informed about the clinical trial prior to enrollment in detail by investigators. A detailed conversation between a medical member of the trial team and the patient will take place discussing all information. These information will be shared in oral as well as in written form. Potential participants will only be asked to sign and personally date two copies of the consent form upon clarification of all questions. Subsequently, one copy of the patient information/consent form will be handed to the participating individual; the second copy will remain at the study site.

### Description of the investigational product

The investigational medicinal product consists of isolated and purified autologous pancreatic islets and will be manufactured, tested and released patient-related under GMP conditions according to the German Drug and Drug Substance Manufacturing Regulation (Arzneimittel- und Wirkstoffherstellungsverordnung, §16) under the responsibility of the University Hospital Carl Gustav Carus at the Technical University of Dresden.

The pancreatic islets are isolated and purified from the patient’s own pancreatic tissue according to §13 Medicines Act (AMG). The pancreatic resection is performed in accordance with §20b AMG. The isolation process takes place after preparation of the tissue (liberation of surrounding fatty/binding tissue) by distension with an enzyme mixture (collagenase, neutral protease, DNAse) via the pancreatic duct and initiation of the enzymatic digestion process by heating to 35°C and mechanical support. After release of the intact islet cell clusters from the surrounding tissue, the digestion process is stopped by cooling the system and adding albumin-containing medium. Subsequently, the tissue suspension is washed several times, and separation of islet cell clusters and exocrine tissue components is achieved by density gradient centrifugation. The purified islet preparation is transferred to transplantation medium after repeated washing steps. The process is accompanied by defined in-process controls. 

### Treatment regimen

In the control arm, oncological PD is performed as pylorus-preserving pancreatic head resection (PPPD) or as classic Whipple procedure with the only requirement for the form of reconstruction being that the pancreatic reconstruction must be to the jejunum. In the intervention arm, oncological total pancreatectomy is performed. This procedure may or may not include simultaneous splenectomy/partial gastric resection, depending on the situs. In the case of total pancreatectomy, application of the investigational drug then follows once after the above-mentioned islet cell isolation and purification steps. Multiple or re-transplantation is not intended in the context of this study. The extension of the surgical procedure (e.g., oncological reasons) is explicitly not limited by the study protocol.

IAT is performed under general anesthesia at the end of the pancreatic resection. For this, portal vein is accessed and punctured with a three-lumen central venous catheter (CVC). The CVC is advanced into the right portal vein branch. The correct position is checked and documented intraoperatively using ultrasound. A continuous pressure system is connected to the CVC to measure portal vein pressure. After establishing the appropriate infusion systems, the preparation is infused by gravity over a period of approximately 30 minutes. The portal vein pressure is continuously monitored. In case of a pressure increase of >50%, the infusion is slowed down or paused and continued after normalization of portal vein pressure. In case of an irreversible pressure increase above 100%, the infusion is stopped and the remaining preparation is withdrawn.

All study patients receive standard postoperative interdisciplinary follow-up in the surgical intensive care unit to guarantee specific monitoring of islet function and adjustment of diabetes treatment, if applicable.

### Outcome

The primary endpoint of this study is defined as the time between the day of surgery and the day on which a oncologist decides that the patient is fit to receive adjuvant therapy.

The following points were defined as secondary outcome parameters:

- perioperative morbidity and mortality
- preoperative and postoperative quality of life
- metabolic outcome
- frequency and type of adverse events
- long-term oncologic outcome including time between surgery and actual start of chemotherapy

### Description of trial visits

The screening visit marks the start of the study. At this visit, each potential participant will be comprehensively informed about the clinical trial prior to enrollment. The information is provided in-person by a medical member of the trial team and in printed form in a detailed patient information sheet. At this visit, inclusion and exclusion criteria will be reviewed, and demographic data, medical history, and secondary diagnoses will be collected if the patient gave informed consent. In addition, participants will undergo a physical examination (including ECG) and laboratory testing will occur, as well as recording of vital signs and body weight.

The following data will be acquired:

- demographic data (sex, age, body weight, height, BMI)
- medical history, secondary diagnoses, concomitant medication
- diabetes history, diabetes aids
- glycemic parameters (blood glucose determination, HbA1c, C-peptide, fructosamine)
- vital signs (pulse, blood pressure, temperature, body weight)
- physical examination results
- EORTC QLQ-C30/Pan26 questionnaire [20]
- ECG
- blood and urine examination
- pregnancy test
- tumor markers CA19−9 and CEA

**Fig 2:**
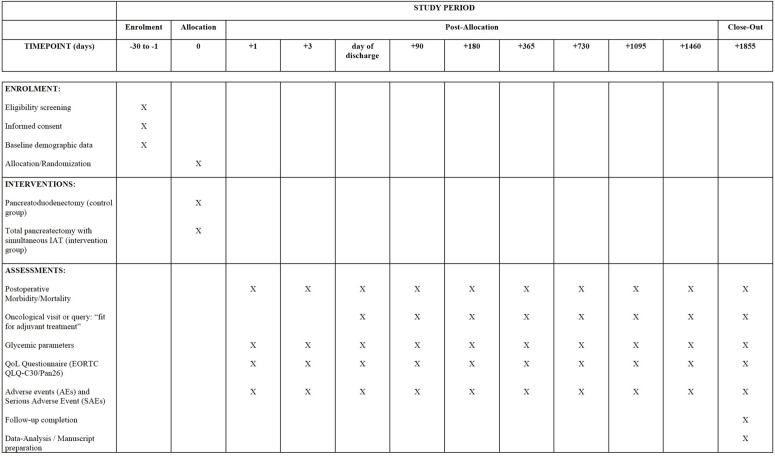
Standard Protocol Items: Recommendations for Interventional Trials (SPIRIT) figure for participant timeline. Abbrevations: IAT, intraportal islet autotransplantation; QoL, quality of life.

All other visits will be performed postoperatively. A total of 10 postoperative visits are planned. The participant timeline is shown in the corresponding SPIRIT figure (Fig 2). A detailed visit schedule and all data collected are attached in supplementary materials (S1 Table).

### Data management, statistics and quality assurance

#### Data management and quality assurance.

The data obtained in this clinical trial will be documented in an eCRF based on the study software MACRO 4.8 (or higher). The eCRF will be generated by Coordination Center for Clinical Trials Dresden (KKS Dresden). The correctness of the documented data will be checked by reconciliation of the source data with the documentation by the monitor of the KKS Dresden according to the specifications of the monitoring plan. In case of discrepancies, queries will be made in the eCRF and will be answered by authorized members of the review group. The eCRF itself contains range, validity, and consistency checks, so that potential errors can already be detected by the database during data entry. The Coordination Center for Clinical Trials Dresden will check the entered data according to the data management plan and will issue appropriate queries in case of discrepancies. Upon completion of the check, the database will be closed after all data has been entered and queries have been resolved. All data management processes will be documented and the documentation will be stored in the TMF.

#### Reporting adverse events.

The safety of both treatment methods is verified by documenting all AEs and SAEs that occur.

The frequency and type of (S)AEs are recorded according to the Common Terminology Criteria for Adverse Events (CTCAE; version 5.0, 2017). Of particular interest here are (serious) adverse events that can at least probably be attributed to the transplant.

The criteria for assessing causality correspond to the WHO-UMC criteria. These parameters are checked as part of the questioning and clinical examination of the patient during each visit after intervention/transplantation. 

#### Statistical methods.

The data collected within this exploratory trial will provide the basis for a subsequent confirmatory controlled study. Therefore, no formal sample size estimation was performed. Assuming an event rate of about 60 percent, a sample size of 30 patients (15 individuals per treatment group) allows for the detection of a hazard ratio of approximately 0.31 (two-sided significance level of 10%, power of 80%). Assuming a drop-out rate of 5%, 16 patients per treatment group will be included. 

The main analysis will be performed after completion of the two-year follow-up period (including data collection and cleaning) in the entire intention-to-treat population. No interim efficacy analyses are planned in this study. All analyses will be performed using SAS 9.4 or higher.

The confirmatory analysis of the primary endpoint and the analysis of the secondary endpoints will be performed according to ICH E9 [21] using the full analysis set (intention-to-treat population) and follow the intention-to-treat principle. The full analysis set will include all randomized patients.

##### Per-protocol population

The per-protocol population will include all patients in the intention-to-treat population without any serious protocol deviation.

##### Safety analysis set

The safety analysis set includes all patients in whom the investigational therapy was used.

For long-term oncologic outcomes (survival analyses), patients lost to follow-up and patients deceased within the observation period will be censored. A per-protocol analysis is planned for sensitivity analysis.

The primary endpoint (time between surgery and day of “fit for adjuvant treatment”) is defined as the interval between day of surgery and day of oncologists decision that patient is fit to start the administration of adjuvant chemotherapy, measured in days. It will be analyzed using Kaplan-Meier estimators (with a two-sided confidence level of 90 percent). Comparison of the primary endpoint between the treatment groups will be performed using the log-rank test. In case of proportional observed hazards in the control and treatment groups, a Cox proportional-hazards model will be used to examine which covariates have an impact on the hazard ratio of the two treatments. Safety analysis will include the frequency of (serious) adverse events in the safety analysis set. Annual safety analyses will be performed in accordance with regulatory requirements. Frequencies and types of adverse events (AE, SAE) will be summarized as relative frequencies (proportions) and, if applicable, the occurrence of specific events (at the patient level) of the two groups will be compared using chi-square test. QLQ-C30/Pan26 questionnaires will be analyzed using a mixed model with the patient as a random factor analysis, including comparison of changes in quality of life over time between the two treatment groups, and respective covariates potentially impacting quality of life within each of the two treatments. Exact Chi-Square test will be used to analyze perioperative morbidity and mortality. Metabolic outcome over time will be analyzed using a mixed model with the patient as a random factor. To analyze the oncologic outcome, a Cox regression model will be used.

## Discussion

POPF is the most relevant PD-associated complication. A recently published ISGPS recommended classification system emphasized a soft pancreatic textur and a small pancreatic duct ≤ 3 mm as the most relevant risk factors for developing POPF [22]. Accordingly, patients at very high-risk of POPF are labeled as ISGPS-D. In these patients, an overall morbidity of 45.9% and in-hospital mortality of 4.1% was reported [23]. These results illustrate that the surgical outcome after PD can have influence on the onlogical long-term outcome. Mackay et al. reported that only one third of patients receive their indicated adjuvant treatment after pancreatic surgery, mostly due operation-associated complications [24]. A previous study had even demonstraded that 67.4% patients with serious POPF-associated complications never delivered adjuvant chemotherapy [25]. These facts leaving a little room for improvement through prophylactic TP. However, a recently systematic review and meta-analysis found no superiority of TP in terms of reduction in short-term mortality and major morbidity compared to high-risk PD [26]. Pancreoprivic diabetes inherently resulting from TP represents the most severe form of diabetes due to complete insulin and glucagon deficiency and consecutively is often associated with severe metabolic lability and frequent hypoglycemia. Furthermore, there is increasing evidence that the quality of metabolic control is strongly correlated with oncologic outcome [27]. Therefore, the results of the recently published PAN-IT trial encourage that total pancreatectomy with simultaneous IAT may become the standard treatment for malignant diseases in candidates for PD, when a high risk of POPF is predicted [28].

The above described product used in this pilot study is already used in standard care at the study site in both allogeneic and autologous settings. The relevant risks relate to the implantation procedure and oncological aspects. Procedure-associated complications are limited to the risk of portal vein thrombosis due to intraportal infusion of islet cells. This risk can be controlled by performing continuous portal pressure measurement during infusion as well as by systemic anticoagulation. Overall, this risk is considered to be low.

A potential risk of IAT in patients with pancreatic malignancy is the transfer of dysplastic cells and induction of liver metastases by intraportal transplantation. During the islet isolation process, endocrine cellular portions of the pancreas are enriched, but complete elimination of ductal cells cannot be guaranteed. However, studies from Balzano *et al.* demonstrated that the oncologic outcome after IAT in patients with malignant disease is not worse compared with patients treated according to standard protocols [10,29]. Moreover, in the case of standard pancreatic head resection, pancreatic parenchyma with potentially dysplastic cells remains in situ. Therefore, the purification and thus significant reduction of exocrine/ductal fractions performed in the course of islet isolation and transplantation can be considered equivalent from an oncological point of view with regard to the amount of potentially malignant cells remaining in situ.

The XANDTX pilot trial will aim to provide feasibility and safety data that will generated a hypothesis and basis for a subsequent confirmatory controlled study that has the potential to provide practice changing data.

## Supporting information

S1 Table Detailed visit plan. (DOCX)

S1 File SPIRIT checklist. (PDF)

## Abbreviations

AMG: Arzneimittelgesetz (German Medicinal Products Act)
BMI: body mass index
CRF: case report form
CVC: central venous catheter
ECG: electrocardiogram
eCRF: electronic case report form
FRS: fistula risk score
IAT: islet autotransplantation
GCP: good clinical practice
PD: pancreatoduodenectomy/pancreaticoduodenectomy
PPPD: pylorus-preserving pancreaticoduodenectomy
POPF: postoperative pancreatic fistula
QoL: quality of life
TMF: trial master file
TP: total pancreatectomy.
