# Supplementary material for: Pancreatoduodenectomy versus total pancreatectomy and simultaneous intraportal islet autotransplantation for periampullary cancer at high-risk of postoperative pancreatic fistula (XANDTX-trial): Protocol of a randomized controlled pilot trial
Source: PLoS One. 2025 Jul 28;20(7):e0327949. doi: 10.1371/journal.pone.0327949 (PMC12303278; doi:10.1371/journal.pone.0327949)
Supplement: S1 Table — (DOCX) [file pone.0327949.s001.docx]

|  | screening visit | intervention | visit 1  (POD 1) | visit 2  (POD 3) | visit 3  (day of discharge) | visit 4  (3-month FU) | visit 5  (6-month FU) | visit 6  (12-month FU) | visit 7  (24-month FU) | visit 8  (36-month FU) | visit 9  (48-month FU) | visit 10  (60-month FU) |
| --- | --- | --- | --- | --- | --- | --- | --- | --- | --- | --- | --- | --- |
| Informed consent | x |  |  |  |  |  |  |  |  |  |  |  |
| In-/exclusion criteria | x | x |  |  |  |  |  |  |  |  |  |  |
| Demographic data | x |  |  |  |  |  |  |  |  |  |  |  |
| Diabetes history/co-morbidities | x |  |  |  |  |  |  |  |  |  |  |  |
| Vital signs | x | x | x | x | x | x | x | x | x | x | x | x |
| Pregnancy test | x |  |  |  |  |  |  |  |  |  |  |  |
| Concomitant medication | x |  | x | x | x | x | x | x | x | x | x | x |
| Blood tests^1^ | x |  | x | x | x | x | x | x | x | x | x | x |
| Glycemic parameters^2^ | x |  | x | x | x | x | x | x | x | x | x | x |
| Adjustment of diabetes therapy | x |  | x | x | x | x | x | x | x | x | x | x |
| Diabetes training | x |  | x | x | x | x | x | x | x | x | x | x |
| Adherence^3^ | x |  | x | x | x | x | x | x | x | x | x | x |
| Operation |  | x |  |  |  |  |  |  |  |  |  |  |
| Randomization |  | x |  |  |  |  |  |  |  |  |  |  |
| Tumor screening (CT/MRI)^4^ | (x) |  |  |  |  |  | x | x | x | x | x | x |
| i.v. glucose tolerance test^5^ |  |  |  |  |  |  | x | x | x | x | x | x |
| QoL Questionnaire (EORTC QLQ-C30/Pan26) | x |  |  |  |  | x | x | x | x | x | x | x |
| Adjuvant therapy |  |  |  |  |  | x | x | x | x | x | x | x |
| Adverse events (AEs) and Serious Adverse Event (SAEs) |  |  | x | x | x | x | x | x | x | x | x | x |
| Oncological visit |  |  |  |  | x |  |  |  |  |  |  |  |

^1^ Blood samples (blood cell differential count, ALAT, ASAT, yGT, AP, bilirubin, creatinine, urea, C-reactive protein, procalcitonin, lipase, amylase, CA 19-9, CEA. Additional parameters according to the clinical course and medical necessity)

^2^ Glycemic parameters (glucose, glucagon, HbA1c, C-peptide, fructosamine; after IAT: continuous glucose monitoring data)

^3^ Adherence concerning diabetes treatment

^4^ CT scans that are not older than 2 months at the time of screening are accepted

^5^ only for patients in intervention group

Abbrevations: CT, computed tomography; FU, follow-up; i.v., intravenous; MRI, magnetic resonance imaging; POD, postoperative day; QoL, quality of life
